# Measurement of ^90^Sr in Marine Biological Samples

**DOI:** 10.3390/molecules27123730

**Published:** 2022-06-09

**Authors:** Fangfang Deng, Feng Lin

**Affiliations:** Laboratory of Marine Ecological Environment Early Warning and Monitoring, Third Institute of Oceanography, Ministry of Natural Resource, 184 Daxue Road, Xiamen 361005, China; dengfangfang@tio.org.cn

**Keywords:** measurement of ^90^Sr, marine biological samples, solvent extraction

## Abstract

Strontium-90 (^90^Sr) is one of the most hazardous radionuclides, and it contributes to radiation exposure by ingestion. The routine determination of ^90^Sr in marine biological samples is highly desirable given the development of the nuclear power industry. A fast, simple, and low-detection-limit method was developed for the measurement of ^90^Sr in marine biological samples based on determining ^90^Y by means of coprecipitation and solvent extraction with bis-2-ethylhexyl-phosphoric acid (HDEHP) in *n*-heptane. The interfering ^210^Bi is removed using Bi_2_S_3_ precipitation. The separation and purification of eight samples per day can be accomplished through this method. The detection limit of ^90^Sr for this method is 0.10 Bq/kg (ash weight). The radiochemical procedure was validated by fitting the decay curve of the sample source and by the determination of ^90^Sr standards.

## 1. Introduction

Strontium (Sr) is an alkaline earth metal found in the environment. There are 27 Sr isotopes, including 4 stable isotopes and 24 radioactive isotopes [1]. Among the radioactive isotopes, the most important, ^90^Sr, has a long biological half-life (approximately 7 years) and radioactive half-life (28.9 years). In addition, ^90^Sr has high radiotoxicity because its metabolism is similar to that of calcium [2]. More than 99% of ^90^Sr accumulates in bone, teeth, and bone marrow after entering organisms and has a residence time of >10 years. As the daughter nuclide of ^90^Sr, yttrium-90 (^90^Y) emits high-energy beta particles, thereby increasing the risk of bone cancer through its accumulation in bone tissues. ^90^Sr can result in great external radiation doses to humans and other living things. However, internal radiation doses can also occur by the ingestion of contaminated food. The sources of ^90^Sr in the marine environment are global fallout from nuclear weapon tests conducted during the 1950s and 1960s [3] and local contamination from nuclear power plant accidents [4].

Since the Fukushima Daiichi nuclear power plant (FDNPP) accident that occurred on 11 March 2011, various radioactive materials (e.g., ^89^Sr, ^90^Sr, ^131^I, ^134^Cs, and ^137^Cs) have been released directly into the sea or through the atmosphere [5,6]. Three years after the accident, the short-lived radionuclides ^131^I (T_1/2_ = 8 days) and ^89^Sr (T_1/2_ = 40 days) were no longer detected, whereas the long-lived radionuclides ^90^Sr and ^137^Cs (T_1/2_ = 30.1 years) were still detected. It was reported that approximately 1 PBq of FDNPP-derived ^90^Sr was released into the Pacific Ocean in the form of highly radioactive wastewater [7], whereas the amount of the released ^137^Cs has been estimated to range from 1 to 3.5 P Bq [8,9]. Recently, an abnormally high value of ^90^Sr above 10^7^ Bq/m^3^ was reported in Fukushima radioactive wastewater treated by Advanced Liquid Processing Systems for the removal of artificial radionuclides before discharge into the Pacific Ocean [10]. In addition, excess radiation doses caused by elevated ^90^Sr activity may be induced in humans and marine biota by the ingestion of contaminated seafood [11]. However, far less information is available on the distribution of ^90^Sr in marine environments after the Fukushima nuclear accident (FNA) than on that of ^137^Cs [7], mainly because the analytical methods for determining ^90^Sr generally require tedious sample separation and purification steps, resulting in long analytical times. For example, analytical methods based on β spectrometry usually require an equilibrium time of more than two weeks and a total procedure time of three weeks or more.

The most commonly used measuring instruments to determine ^90^Sr are based on β spectrometry and include the gas proportional counter, liquid scintillation counter (LSC), and Cerenkov counter. To eliminate the influence of the matrix when determining ^90^Sr in different sample matrixes, Sr or Y should be separated from the sample matrix.

There are four frequently used methods for ^90^Sr separation and purification, including coprecipitation, ion exchange, solvent extraction, and extraction chromatographic techniques [12,13].

The fuming nitric acid method was the first to be used and has been applied widely to separate Ca from Sr. Although the method is tedious, wastes much time, and uses hazardous chemicals, this is still the method selected to treat large samples containing a substantial amount of stable Sr.

Solvent extraction is a method of extracting radionuclides from acid solution with conventional extractants. Di-(2-ethylhexyl) phosphoric acid (HDEHP) and tributyl phosphate (TBP) are widely used to separate ^90^Y from ^90^Sr. The application of a mixture of HDEHP and toluene to selectively extract ^90^Y from acid solution has been reported [14]. This method is relatively simple and fast.

Recently, a simple and fast chromatographic extraction method using Sr resin has been successfully used for environmental samples, but the technique has relatively high minimum detection limits (MDLs). A rapid analytical method was reported for analyzing 1 L seawater with a sample preparation time of less than 4 h and MDLs of 0.18 and 0.11 Bq L^−1^ for LSC and Cerenkov counting, respectively, with a 60 min counting time [15]. The coprecipitation and Sr resin methods were applied to the analysis of 40 mL milk samples, and an MDL of 2.83 ± 0.3 Bq L^−1^ with an obtained counting time of 30 min [16]. The time required for the whole analysis and measurement procedure was only 5 h for 12 samples. However, these methods with high MDLs are unsuitable for the measurement of ultralow ^90^Sr concentrations during routine monitoring. In addition, the cost of direct ^90^Sr determination using Sr resin is high when many samples with a high stable Sr content are processed, such as marine biological samples. Tazoe et al. [17] reported a method for the ^90^Sr analysis of seawater samples using DGA resin; however, the sample volume was only 3 L.

With the rapid development of the nuclear power industry, especially after the FDNPP accident, the routine monitoring of ^90^Sr in marine biota has become desired. In this paper, we propose a simple and fast separation scheme for the determination of ^90^Sr in marine biota during routine monitoring that applies coprecipitation and HDEHP liquid extraction methods. The separation and purification of eight samples took only 5 h. In addition, this method is very economical due to the lack of expensive reagents and consumables used. To validate the proposed scheme, we fitted the decay curve of the sample source and applied it to ^90^Sr standards. The method in this paper can be accurately used to measure the activity of ^90^Sr in marine biological samples. The results are of great significance for assessing the impacts of FNA in terms of both ^90^Sr activity in marine biota and the radiation doses to marine species and humans.

## 2. Materials and Methods

### 2.1. Site Description

The sampling method and sampling sites were previously described [11]. In brief, we collected nekton species in the Northwest Pacific, including squid (*Ommastrephes bartramii*), snake mackerel (*Gempylus serpens*), pelagic stingray (*Pteroplatytrygon violacea*), flying fish (*Cheilopogon pinnatibarbatus*), rough triggerfish (*Canthidermis maculatus*), and Japanese amberjack (*Seriolina nigrofasciata*), between May and June 2012. The species sampled in the Taiwan Bank Fishing Ground included grouper (*Epinephelus awoara*), pufferfish (*Takifugu reticularis*), bream (*Scolopsis vosmeri*), and wrasse (*Choerodon azurio*).

### 2.2. Reagents and Apparatus

All reagents were prepared from electroindustrial grade components. A ^90^Sr-^90^Y certified reference solution was purchased from Physikalisch-Technische Bundesanstalt (PTB) (Braunschweig, Germany). Count rates were measured using a gas-flow β counter (MPC9604) purchased from Ortec, Inc. (Easley, SC, USA) with a background count rate of approximately 0.6 min^−1^.

### 2.3. Sample Pretreatment

**Figure 1:**
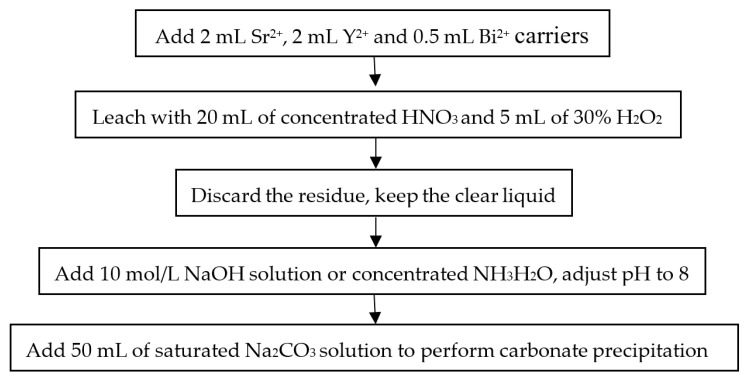
Pretreatment procedure for the marine biota samples.

The main purpose of sample pretreatment is to release ^90^Sr and ^90^Y from the sample matrix and concentrate them in a small amount of liquid solution for the separation and purification of ^90^Y. After being defrosted and weighed, the samples were dried at 105 °C in an oven and transferred into a muffle furnace at a temperature of 450 °C until completely ashed. The ash was cooled to room temperature, ground, and weighed [11,18]. Pretreatment procedure for the marine biota samples is shown in Figure 1. Approximately 10 g of sample ash was weighed accurately and transferred to a glass beaker with a volume of 150 mL, and then 2 mL 100 mg/mL Sr^2+^ (Sr(NO_3_)_2_) and 0.5 mL 20 mg/mL Bi^3+^ (Bi(NO_3_)_3_·5H_2_O) carrier were spiked with pipette tips. A weighed aliquot of 20 mg Y^3+^ carrier (Y_2_O_3_) was added to the sample solution to quantify the yield throughout the radiochemical separation and to determine the radiochemical recovery of the method. To extract ^90^Sr and ^90^Y from the nitric acid leaching liquor, the sample was digested on an electric stove for approximately two hours following the addition of 20 mL concentrated HNO_3_ and 5 mL 30% H_2_O_2_. The sample solution was filtered, the residue was discarded when the temperature dropped to room temperature, and the pH was adjusted to 8 by adding 10 mol/L NaOH solution or concentrated NH_3_·H_2_O. Carbonate precipitation was formed through the addition of 50 mL of saturated Na_2_CO_3_ solution to concentrate ^90^Sr and ^90^Y.

### 2.4. Separation and Purification of ^90^Y

**Figure 2:**
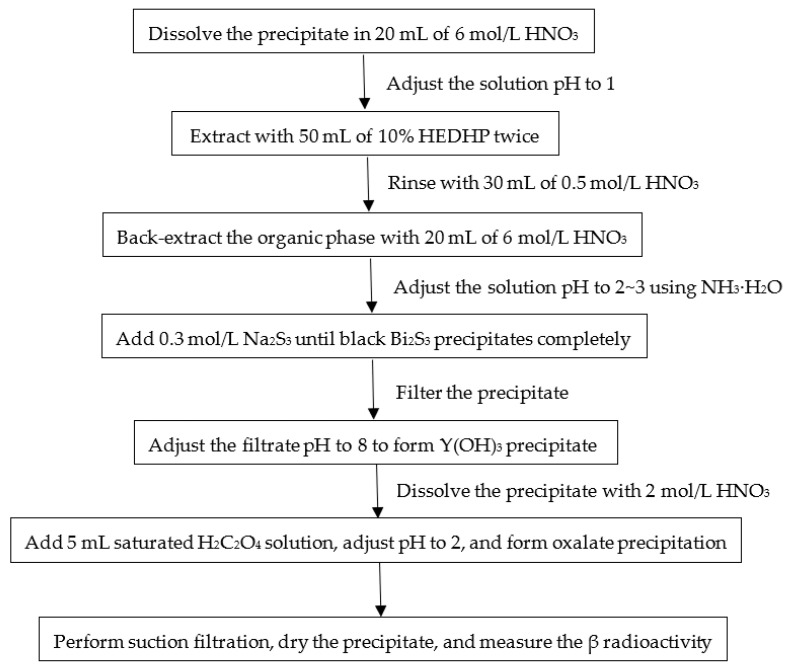
Separation and purification procedure of ^90^Y in marine biota samples.

Separation and purification procedure of ^90^Y in marine biota samples is shown in Figure 2. After filtration, the carbonate precipitate was dissolved in 6 mol/L HNO_3_ with a volume of approximately 20 mL. The pH was adjusted to 1 with concentrated NH_3_·H_2_O. Yttrium in the solution was extracted twice using 50 mL of HDEHP:*n*-heptane solution with a volume ratio of 1:9 to remove interfering elements such as Ca and Sr. The organic HDEHP phase was washed with 30 mL of 0.5 mol/L HNO_3_ to prevent emulsification of the solution, and Y was back extracted twice from the organic phase using 20 mL of 6 mol/L HNO_3_. The time was recorded as the chemical separation time t_1_. The pH of the solution was adjusted to 2–3 using NH_3_·H_2_O. Bi_2_S_3_ precipitate was formed with the addition of 1 mL of 0.3 mol/L Na_2_S solution to remove ^210^Bi, and the sample was then filtered. The pH value of the filtrate was adjusted to 8–9 using concentrated NH_3_·H_2_O to further remove interfering elements and to form the Y(OH)_3_ precipitate. After filtration, the filtrate was discarded, and the Y(OH)_3_ precipitate was dissolved in 2 mol/L HNO_3_. The Y_2_(C_2_O_4_)_3_ precipitate was formed by adding 5 mL of saturated H_2_C_2_O_4_ solution, and the pH was adjusted to 2 through the addition of NH_3_·H_2_O. After filtration, the Y_2_(C_2_O_4_)_3_ precipitate was dried to constant weight, and the recovery of Y was calculated from its weight. Finally, the sample was placed into a gas-flow β counter to determine the amount of ^90^Y. We achieved the separation and purification of 8 samples per day. The activity of ^90^Sr was calculated from the ^90^Y signal according to the following equation:

$$A_{0}=\frac{\left(n_{1}-n_{0}\right)\times e^{\lambda_{1}\left(t_{2}-t_{1}\right)}\times e^{\lambda_{0}\left(t_{1}-t_{0}\right)}}{m\times \varepsilon \times Y_{Y}}\times \frac{\lambda_{1}\left(t_{1}-t_{0}\right)}{1-e^{-\lambda_{1}T}}$$

where *n*_1_ and *n*_0_ denote the β counting rate for the sample and the instrumental background, respectively; *ε* is the counting efficiency; *m* is the mass of the sample; Y_y_ is the chemical yield of Y; *λ*_1_ and *λ*_2_ represent the decay constants of ^90^Y and ^90^Sr, respectively; and *t*_0_, *t*_1_, *t*_2_, and *T* are the sampling time, separation time of ^90^Sr or ^90^Y, detection time of ^90^Y, and time interval for ^90^Y in the instrument, respectively.

### 2.5. Determination of Counting Efficiency

Each probe of the gas-flow β counter was calibrated using 4 parallel samples in sequence, and the counting efficiency was the average of the 4 results. A spiked standard ^90^Sr-^90^Y solution (6.6 Bq/sample) was transferred to a 50 mL centrifuge tube, 1.00 mL of Sr^2+^ and 1.00 mL of Y^3+^ carrier solution were added, and the sample was diluted with 2 mol/L HNO_3_ to approximately 30 mL. The solution was adjusted to a pH of 8 twice with concentrated NH_3_H_2_O and then centrifuged to remove the supernatant and retain the precipitate to separate ^90^Sr and ^90^Y. The precipitate in the centrifuge tube was dissolved with 2 mol/L HNO_3_, and saturated oxalic acid was added at pH ~1. The sample preparation and determination process were the same as those in Section 2.4. The counting efficiency was calculated using the following expression:

$$\varepsilon =\frac{R_{std}-R_{0}}{A_{std}}$$

where *R_std_* is the net count rate of spiked ^90^Sr-^90^Y standard solution (cps); *R*_0_ is the counting time for the background count rate (cps); and *A_std_* is the activity of the spiked ^90^Sr-^90^Y standard solution (Bq).

## 3. Results and Discussion

### 3.1. Counting Efficiency Results

The parallel results for the detection efficiency of each probe are shown in Table 1. The relative standard deviation was less than 3%, which indicated that the instrument has good stability.

### 3.2. ^210^Bi Removal

**Figure 3:**
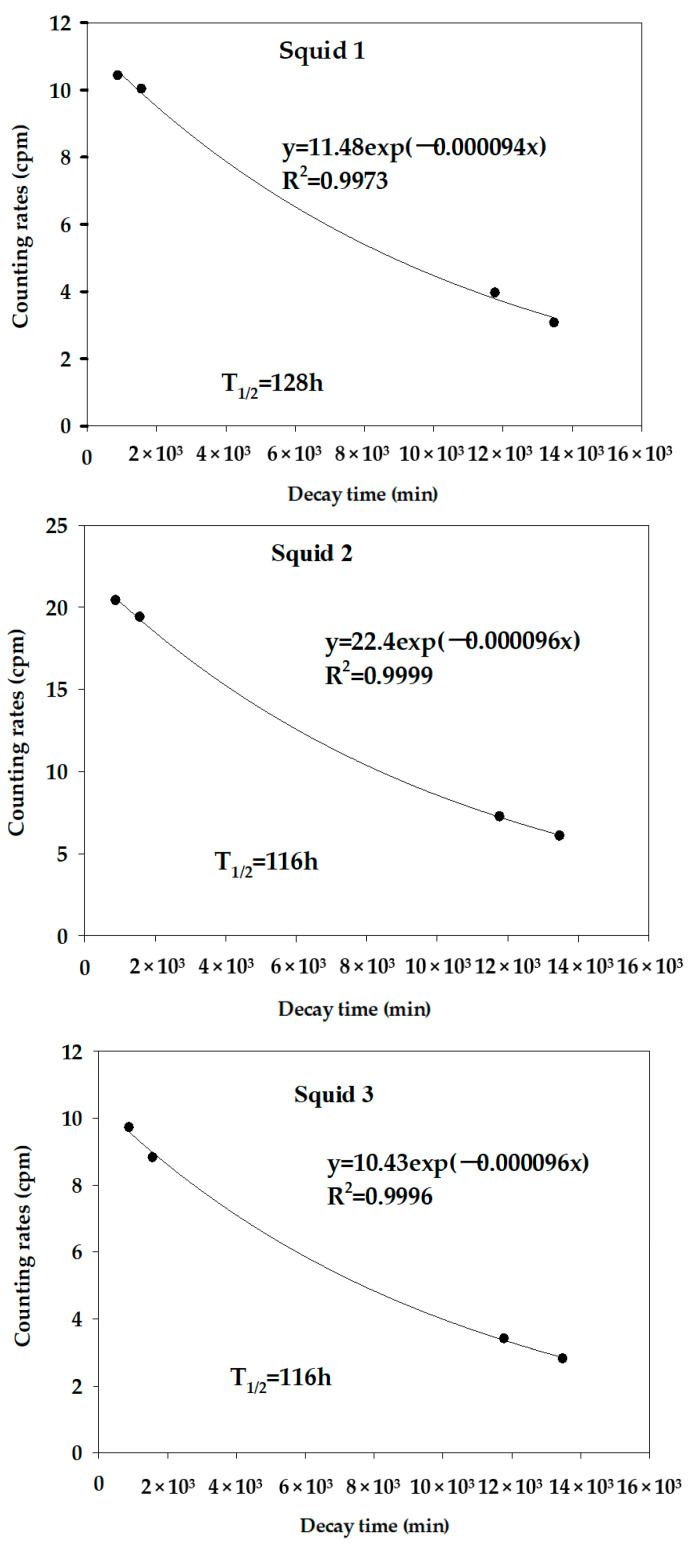
Decay curves of squid samples after solvent extraction with HDEHP.

After the pretreatment, the subsequent separation and analysis steps for ^90^Sr in the marine biota samples were similar to those described for seawater samples [19,20,21]. Our results showed that the activity of ^90^Sr in the three squid samples was abnormally high (Table 2). To determine the reason for the high ^90^Sr activity, we counted the samples using low-background α/β counters at different time intervals. The half–life of the β emitter was found using an exponential decay curve (Figure 3). The average half-life (120 h) corresponded to the β emitter ^210^Bi.

After the interference of ^210^Bi was identified, we designed a procedure for removing ^210^Bi from the chemical mixtures. Deng et al. [22] applied the precipitation of Bi_2_S_3_ to remove ^210^Bi from sediment. The specific operation steps were as follows: the pH of the back-extracted solution was adjusted to 2–3 using NH_3_·H_2_O, and then 1 mL of 0.3 mol/L Na_2_S_3_ solution was added to form a Bi_2_S_3_ precipitate. The decontamination factor of ^210^Bi was higher than 10^3^ when the sediment was treated by Bi_2_S_3_ precipitation [22]. In this paper, we used the precipitation of Bi_2_S_3_ to remove ^210^Bi in marine biological samples similar to sediment, and the final method is described in the experimental section (Section 3).

### 3.3. Verification of the Method

First, the method detection limit was validated for each sample in terms of the MDL, defined as follows [23]:

$$MDL=\frac{50\;\left\{1+\sqrt{1+b/12.5}\right\}}{t\times \varepsilon \times M\times R}$$

where *b* is the total background count and t is the background counting time (in seconds), which, in this case, is the same as the sample counting time. The calculated *MDL* was 0.10 Bq/kg _ash weight_ (assuming 10 g of sample ash) or 10 mBq/sample. In recent years, an increasing number of studies have focused on the application of extraction chromatography in ^90^Sr determination [15,16,24]. Table 3 shows the MDLs for the determination of ^90^Sr in samples with different media; it is evident that the method presented in this paper has a relatively low detection limit.

**Figure 4:**
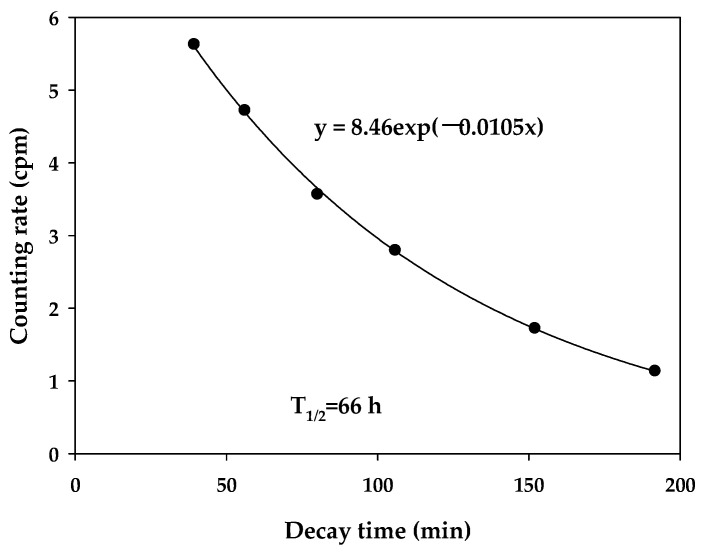
Decay curve of the squid samples based on the described analytical procedure.

To validate the modified method, five squid ash samples (squid 1, squid 2, squid 3, squid 4, and squid 5) were digested, separated, and purified according to the analysis method in this paper. Finally, the acid solutions of the five samples were combined to prepare one sample source. We counted the sample source at different time intervals. The half-life of the β emitter was found using an exponential decay curve (Figure 4). The half-life (66 h) corresponded to the β emitter ^90^Y. We also prepared two standard samples by adding a ^90^Sr standard solution (approximately 87 Bq) to the nekton ash samples (the background activity was less than 0.01 Bq) and measured them using the modified method. The results are shown in Table 4. The measured ^90^Sr data were consistent with the activity of the ^90^Sr standard within the experimental error, suggesting that the modified method is applicable.

To further ensure the reliability of the method, we used it to analyze nekton samples collected from the North Pacific between May and June 2012. The ^90^Sr data have been reported in detail by Men et al. [11]. ^90^Sr in squid falls in the range of nd–0.052 Bq/kg (fresh weight). The ^90^Sr activities in the pelagic stingray and rough triggerfish were 0.01 Bq/kg and 0.055 Bq/kg, respectively. These data were comparable to historical measurements [25,26]. The activities of ^90^Sr in grouper, bream, and wrasse were slightly higher than those of nekton species in the North Pacific but were still within the background level range of the Chinese coastal area.

## 4. Conclusions

When ^90^Sr in marine biota samples was separated and purified using the HDEHP extraction method without added sodium sulfide, it was found that the levels of ^90^Sr in the samples were abnormally high. By fitting the sample β signals, it was found that ^210^Bi interferes with the measurement of ^90^Sr. The ^90^Sr results after Bi_2_S_3_ precipitation in the spiked samples and the nekton samples collected from the Northwest Pacific show that the method is accurate and reliable. Moreover, a low detection limit of 0.10 Bq/kg (_ash weight_) for ^90^Sr was obtained. Finally, the method proposed in this work is especially suitable for marine biota safety monitoring due to the effective sample pretreatment method.

## Figures and Tables

**Table 1:** Parallel results of counting efficiency.

Probe Number	Solution 1 (%)	Solution 2 (%)	Solution 3 (%)	Solution 4 (%)	Counting Efficiency (%)	RSD (%)
MPC9604 (A)	50.6 ± 0.4	52.8 ± 0.8	53.5 ± 0.9	51.8 ± 0.8	52.2 ± 1.3	2.4
MPC9604 (B)	51.0 ± 0.5	51.4 ± 0.7	50.9 ± 0.8	50.9 ± 1.0	51.1 ± 0.2	0.5
MPC9604 (C)	50.6 ± 0.6	50.2 ± 0.5	51.2 ± 1.0	51.9 ± 1.0	51.0 ± 0.7	1.5
MPC9604 (D)	50.2 ± 1.8	50.8 ± 0.9	50.1 ± 0.7	50.5 ± 0.8	50.4 ± 0.7	0.6

**Table 2:** Specific activity of ^90^Sr in the squid samples (uncertainties are expressed at k = 1).

Nekton Species	^90^Sr (Bq/kg_(fresh weight)_)
Squid 1	1.37 ± 0.01
Squid 2	2.89 ± 0.02
Squid 3	3.89 ± 0.02

**Table 3:** MDLs of selected radiochemical procedures for ^90^Sr determination in different sample matrices.

Sample Matrix	Main Separation Method	MDL (mBq/Sample)	Reference
Water	Rapid resin column separation	140	[24]
Seawater	Coprecipitation and DGA resin	1000	[15]
Milk	Coprecipitation and Sr resin	113	[16]
Marine biological sample	HDEHP extraction	10	This study

**Table 4:** Results obtained for two standard samples (uncertainties are expressed at k = 1).

Standard Sample	Standard Activity (Bq)	Measured Value (Bq)
Squid ash spiked with ^90^Sr standard	87.07 ± 1.81	84.97 ± 4.73
Snake mackerel ash spiked with ^90^Sr standard	87.28 ± 1.81	88.20 ± 4.96

## Data Availability

Not applicable.
